# Hemophagocytic Lymphohistiocytosis in a Pediatric Lung Transplant Recipient

**DOI:** 10.1111/petr.70232

**Published:** 2025-11-25

**Authors:** Sunil Chickmagalur, Anna Schrader, Dallas Parrish, David Moreno McNeill, Maria C. Gazzaneo, Ernestina Melicoff‐Portillo, Nahir Cortes‐Santiago

**Affiliations:** ^1^ Division of Pulmonology, Department of Pediatrics Baylor College of Medicine Houston Texas USA; ^2^ Division of Pediatric Pulmonology, Department of Pediatrics Texas Children's Hospital Houston Texas USA; ^3^ Division of Critical Care, Department of Pediatrics Baylor College of Medicine Houston Texas USA; ^4^ Department of Pathology & Immunology Baylor College of Medicine Houston Texas USA

## Abstract

**Background:**

Hemophagocytic lymphohistiocytosis (HLH) is a rare complication of solid organ transplantation and is a syndrome of systemic hyperinflammation secondary to dysregulation of the inflammatory response, primarily involving lymphocytes and macrophages. It is often fatal and therefore early recognition and treatment are crucial. Among 11 adult cases of HLH in post‐lung transplant cases found in the literature, only one patient survived.

**Case:**

We report the first known pediatric case of HLH following lung transplantation. The patient, a previously healthy adolescent, developed end‐stage bullous lung disease secondary to acute respiratory distress syndrome (ARDS) and underwent bilateral lung transplantation. Two months posttransplant, he was admitted with an asymptomatic febrile illness of unclear etiology. By day four, evolving multiorgan dysfunction raised concern for HLH. Despite extensive infectious, autoimmune, and malignancy workups, no definitive trigger was identified. Treatment was initiated with dexamethasone monotherapy with subsequent clinical improvement and discharge 1 month later.

**Conclusion:**

Solid organ transplantation appears to raise a patient's risk of developing HLH, although the underlying mechanisms are unclear. Literature review suggests patients are most likely to develop this complication within the first few months of transplantation, and a high index of suspicion must be maintained in those who present with a febrile illness of unclear etiology. Standard HLH treatment protocols may not be applicable to this patient population, and further studies are needed.

**Abbreviations**
ARDS: acute respiratory distress syndrome
ARF: acute respiratory failure
ATG: anti‐thymocyte globulin
BAL: bronchoalveolar lavage
BLT: bilateral lung transplantation
CMV: cytomegalovirus
CT: computed tomography
EBV: Epstein–Barr virus
FDA: food and drug administration
GVHD: graft‐versus‐host disease
HHV‐6: human herpes virus 6
HHV‐8: human herpes virus 8
HLH: hemophagocytic lymphohistiocytosis
MAS: macrophage activating syndrome
MSSA: methicillin‐susceptible *Staphylococcus aureus*

PET: positron emission tomography
PTLD: post transplant lymphoproliferative disease
SOT: solid organ transplantation
TMA: thrombotic microangiopathy
VV‐ECMO: venovenous extracorporeal membranous oxygenation

## 1 Introduction

Bilateral lung transplantation (BLT) is a therapeutic option for children with progressive treatment‐refractory end‐stage lung disease [1]. Posttransplant survival is lower compared to other solid organ transplants (SOT), due in part to the absence of a bronchial artery circulation in the allograft and continuous exposure via inhalation [2]. Complications following BLT are categorized as infectious or noninfectious and tend to occur within defined time frames: the immediate posttransplant period, early phase (1–3 months), and late phase (> 3 months) [1].

**FIGURE 1:**
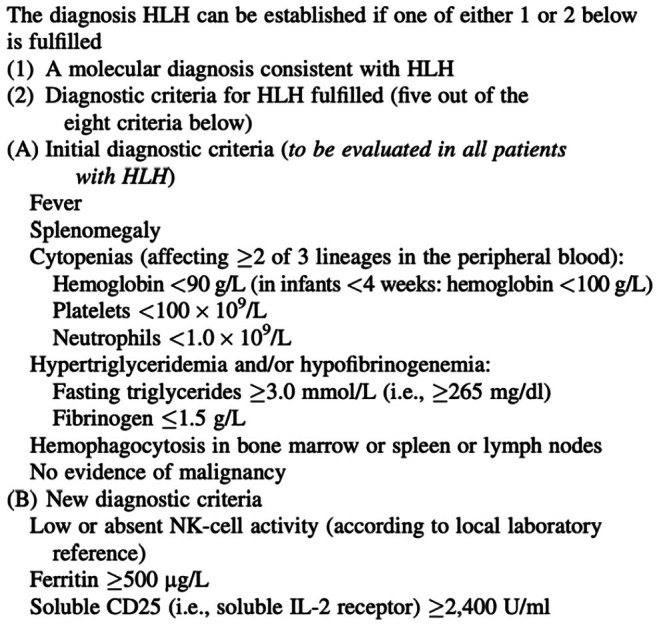
Diagnostic criteria for HLH [4].

Hemophagocytic lymphohistiocytosis (HLH) is a rare and life‐threatening complication following BLT. While HLH has been more frequently described in recipients of other SOTs, only a few adult cases have been reported post‐lung transplantation. HLH results in systemic hyperinflammation driven by persistent activation of cytotoxic lymphocytes and macrophages due to immunedysregulation [3]. It is classified as either primary (genetic) or secondary, with triggers including infection, malignancy, and autoimmune disease. Diagnosis is made based on the presence of an HLH‐related mutation or five or more established criteria (Figure 1) [4]. It is often fatal and therefore early recognition is crucial.

We report the first known pediatric case of HLH complicating lung transplantation: a previously healthy adolescent who developed HLH 2 months post‐BLT. They presented with a febrile illness and subsequently developed multiorgan involvement in the absence of an identifiable etiology.

## 2 Case

A previously healthy 15‐year‐old male presented to an outside hospital in acute respiratory failure (ARF) secondary to suspected bacterial pneumonia, though no infectious etiology was identified. He required invasive mechanical ventilation and ultimately venovenous extracorporeal membranous oxygenation (VV‐ECMO) support for 6 weeks. His slow recovery was complicated by significant parenchymal injury and air leak. After ECMO decannulation and wean to room air, he was discharged but readmitted within 2 days for recurrent respiratory failure and worsening pneumothoraces. Imaging revealed large cystic bullae with minimal healthy lung parenchyma, prompting transfer for lung transplant evaluation.

Since he was previously healthy, the severity of the illness and the degree of subsequent parenchymal damage were out of proportion to what was expected. Pre‐transfer lung biopsy demonstrated organizing fibrinous pleuritis, paraseptal emphysema, and fibrosis. The differential remained broad, though with negative infectious, immunologic, rheumatologic, and genetic workup. The decision was made to cannulate the patient onto VV‐ECMO as a bridge to evaluation for lung transplantation. Though no underlying etiology was found, there was no barrier to transplantation and a suitable offer was received within 24 h.

He underwent bilateral sequential transplantation. Intraoperatively, his immunosuppressive induction included intravenous methylprednisolone, anti‐thymocyte globulin (ATG), and mycophenolate. Donor serologies were positive for cytomegalovirus (CMV) and Epstein–Barr virus (EBV), and bronchoalveolar lavage (BAL) culture grew methicillin‐susceptible *Staphylococcus aureus* (MSSA). The patient's serologies were negative for CMV, positive for EBV, and his explant tissue culture grew *Klebsiella pneumoniae* , a complication from his initial hospitalization.

Post‐operatively, he recovered well and was extubated on day three. Routine surveillance with transbronchial biopsies and serum studies showed no evidence of rejection. He was discharged about 6 weeks after transplantation. His immunosuppression regimen consisted of prednisone, mycophenolate, and tacrolimus, while his prophylactic antimicrobials included azithromycin, valganciclovir, voriconazole, and trimethoprim/sulfamethoxazole (TMP/SMX).

At routine follow‐up 2 months posttransplant, he was febrile to 101.6 degrees Fahrenheit with tachycardia, but without other focal signs or symptoms. He was directed to the emergency department for an infectious workup and admission for antibiotics. His labs were notable for new leukopenia with normal hemoglobin and platelets, mildly elevated creatinine and cystatin C, mildly elevated liver function tests, elevated C‐Reactive Protein, and negative COVID, RSV, and Influenza PCR nasopharyngeal swab. A reflex comprehensive respiratory viral PCR panel and serum blood cultures were pending. Initial chest X‐ray was stable compared to prior.

He remained intermittently febrile (T‐max 103.1) with labs notable for decreasing platelets and worsening transaminitis. Due to his immunosuppressed status, infectious work‐up was expanded to include other viral and atypical organisms (Table 1). Doxycycline was added to his regimen for atypical intracellular bacterial coverage, and computed tomography (CT) imaging was considered. By hospital Day 5, there was worsening pancytopenia and liver function tests (ALT 1492 U/L, AST 1645 U/L, GGT 129 U/L). Additional inflammatory markers were sent and notable for elevated ferritin to 7960 ng/mL, LDH to 1728 U/L, and triglycerides to 299 mg/DL. Fibrinogen, uric acid, and thyroid studies were normal. A CT scan of the chest, abdomen, and pelvis showed small bilateral pleural effusions, mediastinal lymphadenopathy, scattered sub centimeter pulmonary nodules, and a small to moderate pericardial effusion. An echocardiogram confirmed the presence of a pericardial effusion with normal ventricular function and no evidence of vegetation. The hematology/oncology team was consulted with concern for HLH and diagnostic testing included further bloodwork (soluble IL‐2 receptor, CXCL9), bronchoscopy, bone marrow biopsy and lumbar puncture. The differential for underlying etiology for HLH remained broad and included infection, malignancy, rheumatologic disease, genetic disease, and drug‐induced reaction.

**TABLE 1:** Infectious disease testing.

Source	Negative	Positive
Nares	Respiratory viral panel	
BAL	Culture (bacterial, fungal, mycobacteria), Universal PCR (bacterial, fungal, mycobacteria), CMV, EBV, Pneumocystis, Aspergillus Galactomannan, Aspergillus Panel, Mucorales, Legionella, Nocardia, Histoplasma	
Blood	Bacterial culture, Enterovirus, CMB, EBV, Adenovirus, Histoplasma, Cryptococcus, Aspergillus Galactomannan, Typhus Fever Antibody, HSV1, HSV2, Parvovirus B19, HHV‐6, HHV‐7, HHV‐8, Bartonella IgG/IgM, Toxoplasma WB PCR, Toxoplasma IgG/IgM, Strongyloides IgG, Ureaplasma, Karius, West Nile, *Mycoplasma Hominis* , Hepatitis B, Hepatitis C, Hepatitis E, Varicella Zoster, Lymphocytic Choriomeningitis Virus, Parasites Smear, HTLV‐I/II Antibodies, BK Virus, JC Virus, HIV Panel, HIV‐1 RNA	
Bone marrow	Adenovirus, CMV, EBV, Parvovirus B19, fungus culture, mycobacteria culture	HHV‐6 positive: 483
CSF	Cryptococcus, VDRL, EBV, CMV, HHV‐6, Varicella Zoster Virus, JC Virus, Meningitis‐Encephalitis, toxoplasma, West Nile, histoplasma, universal PCR (bacterial, fungal, mycobacteria)	
Urine	Histoplasma, Legionella, Chlamydia, Gonorrhea	
Stool	Gastrointestinal pathogen panel, *Clostridium Difficile* , Ova and parasite, hepatitis E PCR	

**FIGURE 2:**
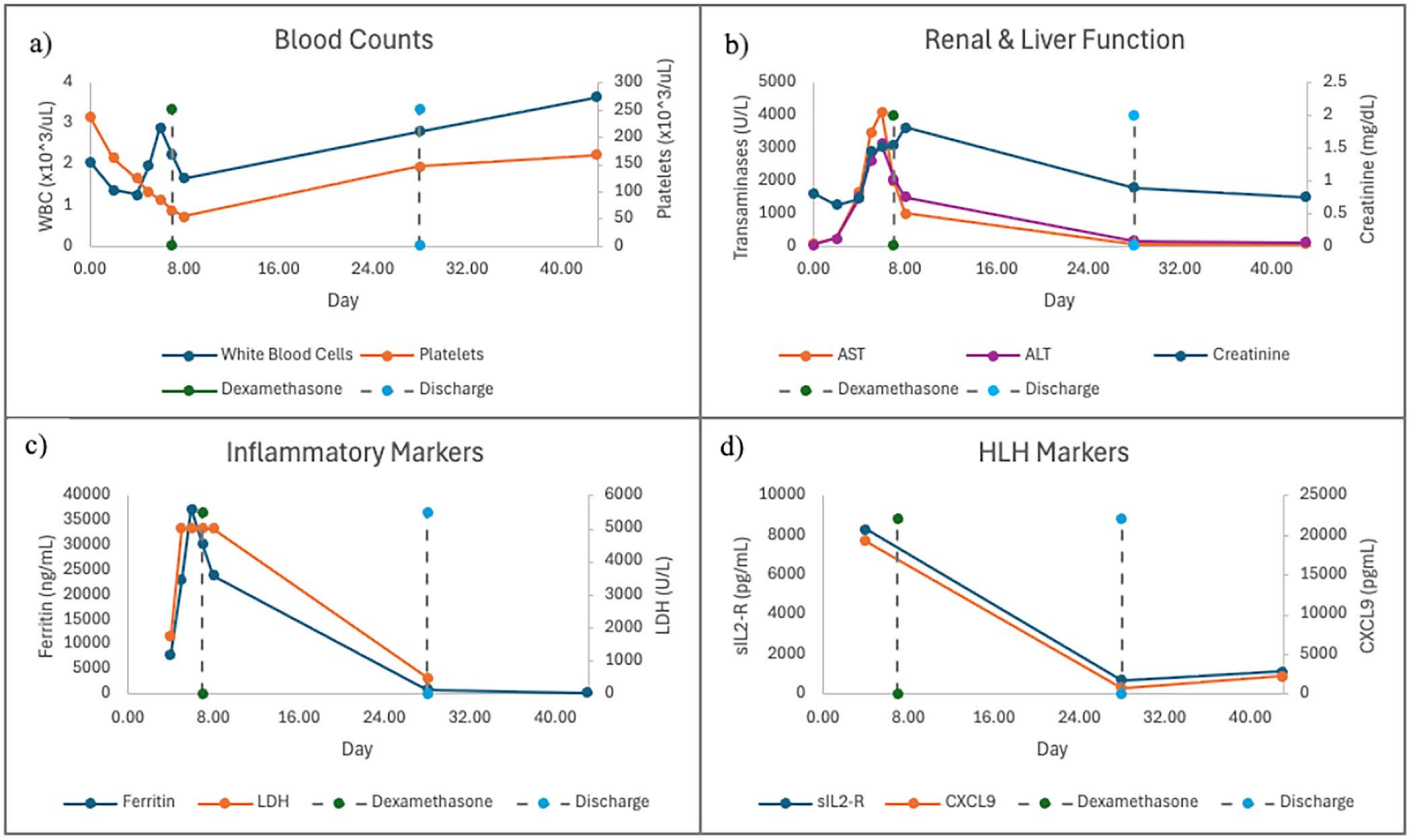
Trend of bloodwork from admission (Day 0) to outpatient follow‐up (Day 43).

Due to rising inflammatory markers (LDH > 5000 U/L, Ferritin of 22 900 ng/mL) and worsening evidence of multiorgan involvement with rising creatinine and liver enzymes, the decision was made to transfer to the ICU post‐operatively (Figure 2c). Bronchoscopy was unremarkable with cytology of lavage fluid composed almost entirely of macrophages, although the presence of frequent hemosiderin‐laden macrophages was noted. He remained febrile but otherwise well‐appearing despite increasing ferritin and worsening transaminitis.

**FIGURE 3:**
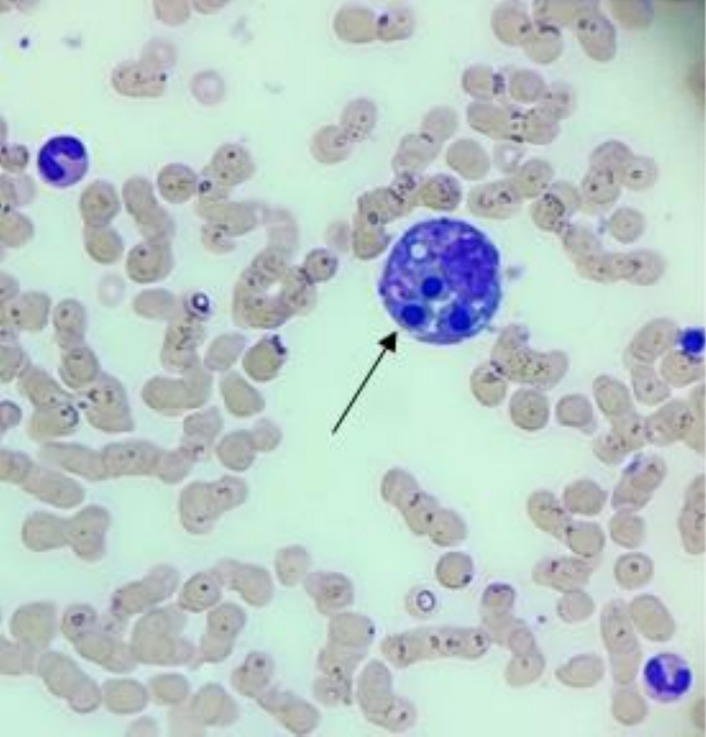
Bone marrow biopsy with presence of histiocyte (macrophage) with hemophagocytosis of nucleated cells.

Bone marrow biopsy showed evidence of a hypocellular marrow and occasional histiocytes with hemophagocytosis, confirming a diagnosis of HLH (Figures 1 and 3). Treatment with steroids was withheld until a positron emission tomography (PET) scan could be done given overall clinical stability. Soluble IL‐2 receptor returned significantly elevated to 8267.1 pg/mL (upper limit of normal 858.2 pg/mL). The lung transplant team confirmed with the organ procurement organization that other transplant recipients from this patient's donor were doing well. The next day, he developed clinically significant hypotension that was responsive to stress dose hydrocortisone. Therefore, he was started on Dexamethasone 10 mg/m^2^ per the HLH‐94 treatment roadmap [5]. His last fever had been the morning before starting steroids, and his liver function tests, and inflammatory markers were now noted to be downtrending after initiation (Figure 2b,c).

Cultures and PCRs from blood, BAL, bone marrow, and CSF were negative for infection (Table 1). Though copies of human herpes virus 6 (HHV‐6) were slightly elevated in the bone marrow, it was not detected in the blood or CSF and therefore deemed insignificant. A PET scan showed no hypermetabolic foci to suggest a lymphoproliferative disease or other malignancy. Chimerism testing evaluating for graft‐versus‐host disease was negative. Reanalysis of whole exome sequencing was negative.

He continued to show lab evidence of response to dexamethasone therapy with a plan for his dose to be weaned by half every 2 weeks per the HLH‐94 roadmap [5]. He was discharged after a one‐month admission. He has demonstrated clinical stability as an outpatient despite some fluctuations in laboratory markers and tolerated the scheduled wean of dexamethasone without reactivation of disease (Figure 2d).

## 3 Discussion

Hemophagocytic lymphohistiocytosis (HLH) is a rare systemic inflammatory disorder with a wide range of symptoms making early clinical diagnosis and management a challenge. Clinical presentation is often nonspecific, typically including prolonged fever, hepatosplenomegaly, cytopenia, and elevated inflammatory markers, which can mimic other conditions and delay diagnosis. In posttransplant patients specifically, diagnoses to consider include severe infectious states (sepsis, systemic inflammatory response syndrome (SIRS), multiorgan dysfunction syndrome), posttransplant lymphoproliferative disease (PTLD), thrombotic microangiopathy (TMA), and graft‐versus‐host disease (GVHD), among others [6]. Although the mechanisms underlying HLH are not fully understood, it results from hyperactivation of macrophages and hypersecretion of inflammatory cytokines secondary to impaired cytotoxic T‐lymphocytes and natural killer cells. This cytokine storm results in the clinical and laboratory features of HLH which may culminate in multi‐organ failure [7].

HLH can be classified as primary or secondary HLH based on the underlying etiology. Primary or familial HLH is caused by a genetic mutation and typically occurs in infancy or early childhood, while secondary HLH can result from inappropriate hyperactivation of the immune system from various triggers [4]. Further complicating diagnosis and management is that many HLH triggers (i.e., infection, malignancy) can also mimic HLH, and would be best treated by targeted therapies. Empiric therapeutic immunosuppression could lead to unnecessary morbidity and mortality in these cases, and therefore a broad workup is necessary [8].

A large national retrospective cohort study examined adult patients admitted with a diagnosis of HLH between 2006 and 2019 (16 136 patients) and found that the commonly associated conditions were malignancy (31%), infection (24%), and autoimmune (21%), with organ transplant‐associated cases making up 4% (639 patients) of the study population. Of those 639 cases, 204 patients had undergone bone marrow transplantation and 191 had undergone kidney transplantation, with the remaining 254 listed as “other” [9].

HLH associated with SOT (HLH‐SOT) is rare and research is mostly limited to case reports or series. Given this knowledge gap, a retrospective review was recently conducted for all cases of HLH‐SOT between 1979 and 2023. This review identified 179 adult and pediatric cases, and further breakdown showed most patients underwent kidney transplantation (118 total, 9 pediatric), 44 patients underwent liver transplantation (20 pediatric), and the remaining 17 cases included 10 lung transplantations (0 pediatric). The prevalence of HLH‐SOT ranges from 0.37% to 1.72%, which is higher than the prevalence of secondary HLH in the general population (1/800 000) [6, 10]. In this cohort, the most common trigger for HLH‐SOT was viral infection, with the top four being CMV, EBV, human herpes virus 8 (HHV‐8), and HHV‐6.

A literature review of lung transplant‐associated HLH identified seven case reports or series covering 11 adult patients [11, 12, 13, 14, 15, 16, 17]. These patients ranged from 27 to 68 years old and developed HLH typically within several months of transplantation. An infectious trigger was identified in at least seven of the patients, with EBV being the most common in three patients. Only two of the 11 patients showed resolution of HLH after treatment, but one experienced a fatal infection during the same admission, highlighting the poor prognosis.

Given the paucity of data on HLH‐SOT, the mechanisms for increased incidence of HLH in this immunosuppressed population remain unclear, though there is an increased incidence of common HLH triggers such as infection and malignancy in these patients [2]. Posttransplantation, long‐term survival can be limited by allograft rejection, and therefore lifelong immunosuppression is necessary. Although practice varies from center to center, immunosuppression regimens typically begin with an induction phase followed by maintenance therapy to primarily reduce T‐lymphocyte activation and proliferation, and some agents can reduce B‐lymphocyte proliferation as well (i.e., MMF) [18].

Our patient received ATG as an induction agent followed by a triple therapy maintenance regimen with tacrolimus, MMF, and methylprednisolone. Due to the immunocompromised state of these patients, prophylactic antimicrobials are necessary. Our patient was initially discharged on valganciclovir, voriconazole, and TMP/SMX. There have been single case reports of suspected medication‐induced HLH involving TMP/SMX and MMF, respectively [19, 20]. In the first case, a previously healthy patient developed signs and symptoms of HLH within 2 days of starting TMP/SMX for a perirectal abscess. A broad workup did not find an alternative explanation, and they responded well to dexamethasone monotherapy. In the second case, a patient who had undergone renal transplantation developed concern for HLH 2 months after transplantation with a negative workup. The patient showed partial improvement to high‐dose glucocorticoids but did not experience complete improvement until a few days after discontinuing MMF, thus raising suspicion for MMF‐induced HLH. For our patient, TMP/SMX was discontinued as a possible causative agent, while MMF was discontinued because of profound myelosuppression.

Treatment for HLH is largely driven by the HLH‐94 protocol which was revised in 2004 and typically consists of 8 weeks of dexamethasone, etoposide, and cyclosporine, followed by continuation therapy if needed [4]. This regimen, however, has not been studied in posttransplant patients. Our patient was successfully treated with dexamethasone monotherapy starting at 10 mg/m^2^ for 2 weeks followed by a six‐week taper. They were maintained on tacrolimus rather than switching to cyclosporine given a similar mechanism of action. The retrospective review of 179 cases of HLH‐SOT found that etoposide was only used in 25 cases with a mortality rate of 56% bringing to question its effectiveness, but there have been studies showing improved outcomes with early use of etoposide in patients specifically with EBV‐associated HLH [21, 22].

Although HLH is a syndrome of an inappropriately hyperactive immune system, this review of HLH‐SOT cases also found that reducing the dosage or stopping immunosuppressive therapy (except for steroids and calcineurin inhibitors) or using steroid monotherapy was associated with higher survival rates, potentially because infection is a common trigger of HLH. Our patient was maintained on tacrolimus while starting dexamethasone therapy. MMF was resumed about 4 weeks after discharge once his cytopenias improved, and there were no subsequent concerns for relapse.

There is a growing body of literature evaluating the use of biologic therapies such as Anakinra (IL‐1 antagonist), Emapalumab (interferon gamma antagonist), and Ruxolitinib (janus kinase inhibitor) in both primary and secondary HLH (infection, rheumatologic) due to their broad cytokine modulating abilities [23]. Emapalumab in particular was approved by the Food and Drug Administration (FDA) for use in pediatric primary HLH in 2018, and recently expanded to include macrophage activating syndrome (MAS) related HLH [24, 25, 26, 27]. However, there is very little reported use in adult SOT patients, and none identified in pediatric patients. Further studies are needed regarding SOT patients, specifically for those who are intolerant of or nonresponsive to standard therapies.

## 4 Conclusion

HLH is a rare complication of transplantation, and the prognosis is poor given the degree of systemic hyperinflammation and the risk of multiorgan involvement. Therefore, a high index of suspicion must be maintained for patients presenting with a febrile illness of unclear etiology, especially within the first year of transplantation. To our knowledge, this is the first pediatric case of HLH in a post‐lung transplant patient and adds to the limited existing case reports of this complication in those who have undergone BLT. The favorable outcome highlights the importance of early diagnosis and suggests that modifications to the standard HLH treatment protocol may be more appropriate for transplant patients, although further studies are needed to establish evidence‐based guidelines for treatment in this patient population.

## Data Availability

Data sharing not applicable to this article as no datasets were generated or analyzed during the current study.
